# Research Progress on Pyroptosis in Hematological Malignancies

**DOI:** 10.1007/s11864-023-01119-7

**Published:** 2023-08-28

**Authors:** Tianxin Lyu, Qingsong Yin

**Affiliations:** Department of Hematology, The Affiliated Cancer Hospital of Zhengzhou University & Henan Cancer Hospital, Zhengzhou, 450008 China

**Keywords:** Hematological malignancies, Pyroptosis, Caspase pathway

## Abstract

Pyroptosis is a kind of programmed cell death dependent on the caspase pathway that is different from apoptosis and necrosis. Recent studies have shown that pyroptosis can be involved in the pathological processes of many diseases, such as cancers, atherosclerosis, diabetic nephropathy, and blood diseases. However, the specific mechanisms by which pyroptosis participates in the occurrence and development of hematological malignant tumors still need further exploration. This article reviews the characteristics of pyroptosis and the regulatory mechanisms promoting or inhibiting pyroptosis and discusses the role of pyroptosis in hematological malignant tumors, which could provide ideas for the clinical treatment of such tumors in the future.

## Introduction

**Fig. 1:**
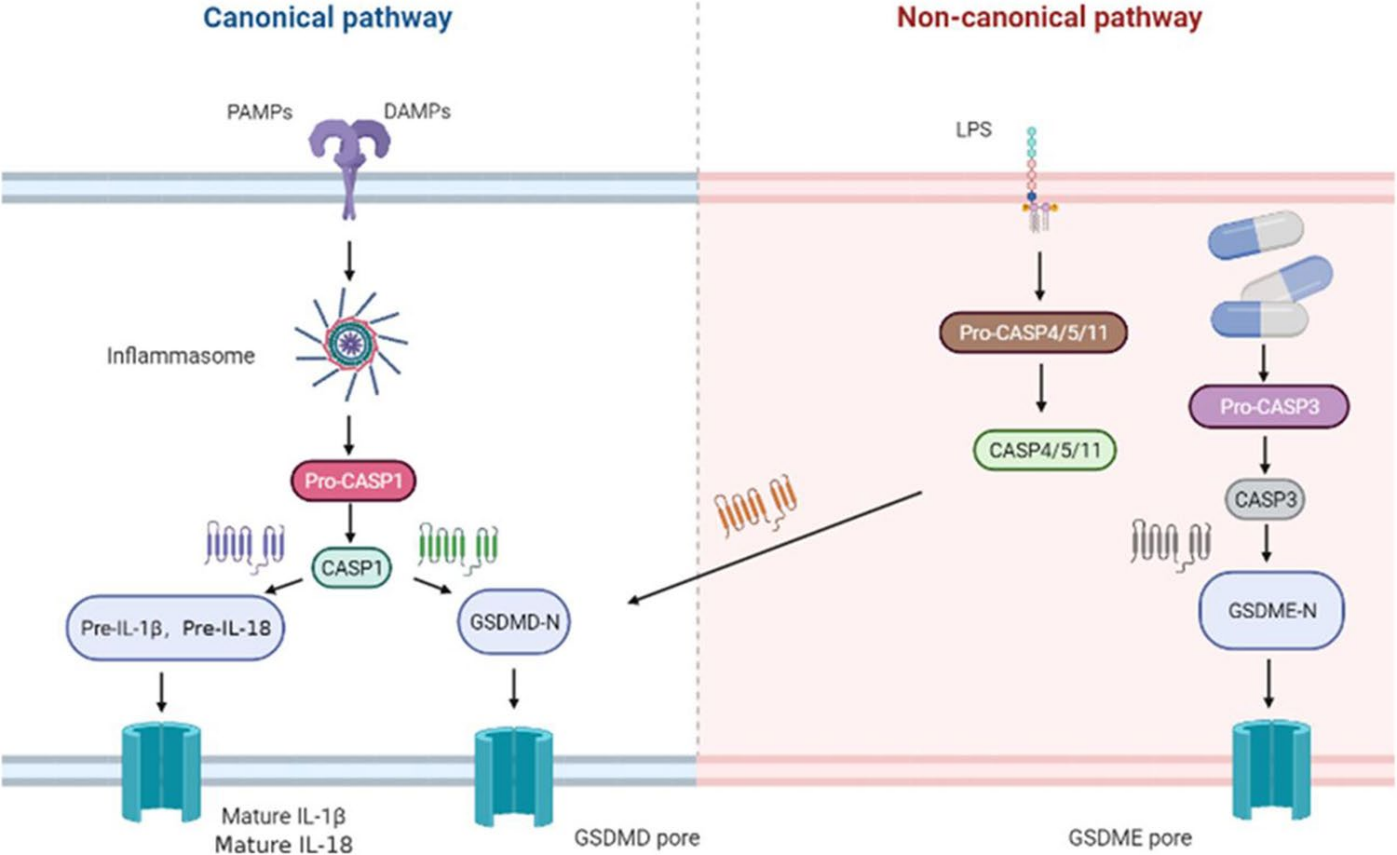
The mechanism of pyroptosis. In canonical inflammatory pathways, PAMPs or DAMPs are identified by inflammasomes, and recombination leads to cleavage and activation of the CASP1 signaling pathway. CASP1 cleaves the inflammatory cytokines IL-1β and IL-18 into mature forms, as well as GSDMD. In noncanonical inflammatory pathways, LPS directly binds to CASP4/5/11, leading to cleavage of GSDMD to produce GSDMD-N, formation of a GSDMD pore, and ultimately pyroptosis. Drugs can cleave GSDME mediated by CASP3, leading to pyroptosis. PAMPS, pathogen-associated molecular patterns; DAMPs, damage-associated molecular patterns; CASP1, caspase-1; CASP3, caspase-3; CASP4/5/11, caspase-4, caspase-5, caspase-11; GSDMD, gasdermin-D; GSDMD-N, GSDMD amino-terminal cell-death domain; GSDME, gasdermin-E; GSDME-N, GSDME amino-terminal cell-death domain.

Pyroptosis is a form of inflammatory cell death dependent on the caspase pathway. It is characterized by the formation of pores in the plasma membrane by members of the gasdermin (GSDM) protein family and was first found to occur in immune cells (such as macrophages, monocytes, and neutrophils) during microbial infection [1••]. Unlike apoptosis, pyroptosis is mainly regulated by inflammation-related caspases, including CASP1, CASP4 (human), CASP5 (human), and *Casp11* (mouse) [2]. Some apoptosis-related caspases, such as CASP3 [3, 4] and CASP8 [5•, 6], also regulate cell death. Caspases mediate the cleavage of members of the GSDM family, such as GSDMD [7] and GSDME [8], which triggers cell death. In canonical inflammatory pathways, pathogen-associated molecular patterns (PAMPs) or damage-associated molecular patterns (DAMPs) are identified by cytoplasmic sensor proteins such as NLRP3 and AIM2, and recombination leads to cleavage and activation of the CASP1 signaling pathway [2], which further promotes cell death through two actions. Caspase-1 cleaves the inflammatory cytokines IL-1β and IL-18 into mature forms, as well as GSDMD [9]. In addition to GSDMD, the cleavage of GSDME (also known as DFNA5) can also activate pyroptosis. Unlike the case in inflammasome-mediated pyroptosis, the cleavage of GSDME is mediated by caspase-3, the executor of apoptosis [10]. Through this mechanism, GSDME plays a role in the transition between apoptosis and secondary pyroptosis. GSDME also forms pores, leading to the release of DAMPs and cytokines [11]. In noncanonical inflammatory pathways, cytoplasmic lipopolysaccharide directly binds to mouse CASP11 or human CASP4/5, leading to inflammasome activation [2]. Finally, activation of the inflammasome causes cleavage of GSDMD and the production of the N-terminal fragment of GSDMD (GSDMD-N), and GSDMD-N mediates pyroptosis through its pore-forming activity on the plasma membrane [12, 13]. (Fig. 1).

Pyroptosis is also observed in tumor cells [14], in which the role of pyroptosis is being increasingly recognized. However, little is known about the role of pyroptosis in hematological malignant tumors. Therefore, an in-depth study of the mechanism of pyroptosis and its relationship with hematological tumors will broaden our understanding of hematological tumors. This paper reviews the mechanism of pyroptosis and summarizes research progress in hematological tumors to provide a reference for relevant follow-up research.

## Research progress in pyroptosis in leukemia

Pyroptosis is a form of programmed cell death that is similar to apoptosis and features karyopyknosis, chromatin DNA fragmentation, and positive TUNEL staining. During cell death, the formation of pores in the cell membrane disturbs the balance of ion gradients, resulting in the release of several intracellular factors and proinflammatory mediators [15, 16]. As a form of cell death, pyroptosis plays a dominant role in preventing the occurrence and development of tumors, especially in leukemia [17, 18].

Evidence has shown that in mouse myeloid cells, small molecular inhibitors of the serine dipeptidase Dpp8/9 can activate the caspase-1 signaling pathway via the inflammasome sensor Nlrp1b, which leads to the cleavage and activation of GSDMD and the formation of a pore in the plasma membrane to mediate cell death and inhibit the progression of acute myeloid leukemia (AML) [19]. Similarly, CARD8 has been identified as a novel inflammasome sensor that can mediate the Dpp8/9 inhibitor-dependent caspase-1 signaling pathway to induce cell death of human myeloid cells. The form of death induced by Dpp8/9 inhibitors in these two types of cells is called pyroptosis. DPP8/9 inhibitors can induce pyroptosis in most human AML cell lines and primary AML samples but not in many other lineages [18]. Interestingly, gene expression analysis showed that upregulated expression of BAY-299 could promote the expression of pyroptosis-related genes, and activation of the caspase-1 or caspase-4, 5, and 11 pathway could induce GSDMD-induced pyroptosis [20]. Alternatively, caspase-3 can be activated to cleave GSDME to induce pyroptosis independent of caspase-1 and GSDMD [3]. In addition to the factors in these two important pathways, GSDMA, GSDMB, and GSDMC also contain a pore-forming domain that can induce pyroptosis. Cytotoxic lymphocyte-derived granzyme A is able to cleave GSDMB and induce tumor cell pyroptosis [21]. GSDMB promotes cell death by enhancing the activity of caspase-4 [22]. However, the exact mechanisms by which GSDMA and GSDMC can be activated and their role in pyroptosis are still unclear. After BAY-299 treatment, the expression of caspase-1, caspase-4, GSDMB, GSDMC, GSDMD, and GSDME in AML cells increases, suggesting that BAY-299 treatment induces apoptosis and triggers pyroptosis. In addition, BAY-299 treatment makes AML cells more sensitive to induced pyroptosis. It is worth noting that the DPP8/9 inhibitors mentioned above are promising for the treatment of AML. Therefore, compared with single treatment, combination with BAY-299 treatment to induce cell death may be a more effective treatment strategy [20]. Through the analysis of candidate genes of primary T cells, we found that the response depends on CARD8-caspase-1-GSDMD signaling. Interestingly, the CARD8-induced pyroptosis pathway can only be activated in a resting state, not under T cell activation. These findings support a correlation between inflammasome signaling pathways and T cells, core components of the adaptive immune system [23]. Curcumin has antileukemia activity. Curcumin can induce the expression of AIM2, IFI16, and the NLRC4 inflammasome in U937 leukemia cells by upregulating the expression of the ISG3 transcription factor complex and then activating caspase-1, promoting the cleavage of GSDMD to induce cell death. In addition, overexpression of exogenous GSDMD induced by lentivirus transfection in K562 cells can enhance the anticancer activity of curcumin, while silencing its expression enhances the resistance of U937 cells to curcumin, suggesting that induction of cell death is a mechanism by which curcumin induces antileukemic effects [24]. Some studies have shown that ardisianone can induce the cleavage of caspase-1, caspase-5, and GSDMD and increase the expression of HMGB1 protein to some extent, indicating a role of ardisianone in inducing pyroptosis in HL60 cells [25]. Many studies have shown that caspase-8 activity and necrotic cell death activated by GSDMD occur via the same signaling pathway [5•, 26]. There is also evidence suggesting that activated caspase-8 can cleave GSDMD, emphasizing the role of caspase-8 in promoting pyroptosis [5•]. Our data show synchronization of the activation of caspase-1, caspase-5, and caspase-8 and the cleavage of GSDMD, suggesting that the caspase activation pathway plays a key role in ardisianone-induced pyroptosis [25]. Application of recombinant Tp92 protein induced the death of the human monocyte cell line THP-1, which was derived from a patient with acute monocytic leukemia, by recognizing cell surface CD14 or TLR2. Stimulation of THP-1 cells with Tp92 protein may induce atypical pyroptosis of THP-1 cells by promoting the caspase-1 pathway [27]. Some studies have shown that necrosulfonamide (NSA), an inhibitor of pyroptosis, can selectively induce highly toxic DNA double-strand breaks and kill AML cells. Reactive oxygen species (ROS) are the key effector substances that mediate NSA toxicity [28]. NSA specifically targets the N-terminal coiled-coil domain of the key programmed necrotic effector mixed lineage kinase domain-like protein (MLKL) to prevent programmed necroptosis and directly disrupt the integrity of the membrane, resulting in necrosis [29, 30]. The active site of NSA is cysteine 86 in human MLKL, but this residue is absent in mouse MLKL. Therefore, NSA specifically functions in humans but does not affect programmed cell death in mice [30]. However, in human and mouse cells, NSA directly binds to the pyroptotic pore-forming protein GSDMD, inhibiting GSDMD oligomerization and pyroptotic cell death [31].

Chronic lymphocytic leukemia (CLL) is another type of hematological malignant tumor characterized by the aggregation of lymphocytes in peripheral blood, bone marrow, the spleen, and lymph nodes. Our research has long focused on research on CLL, but there are limited studies regarding the relationships between CLL and pyroptosis. Salaro et al. [32] reported that the expression of NLRP3 in lymphocytes of patients with CLL is decreased, and CLL cells possibly avoid apoptosis through this mechanism. The expression of NLRP3 is closely related to pyroptosis. However, the mechanism of pyroptosis in CLL still needs further study.

## Research progress on pyroptosis in myelodysplastic syndrome

Myelodysplastic syndrome (MDS) is a heterogenous hematological malignant tumor caused by dyshematopoiesis. MDS bone marrow precursors are characterized by excessive programmed cell death, chromosome abnormalities, and somatic gene mutations and have a tendency to transform into AML [33]. Activation of the NLRP3 inflammasome is a feature of MDS that drives clonal expansion and pyroptosis. Regardless of genotype, MDS hematopoietic stem cells (HSCs) and hematopoietic stem and progenitor cells (HSPCs) overexpress inflammasome proteins and express an activated NLRP3 complex, which can directly activate caspase-1, produce IL-1β, and IL-18 and cause pyroptotic cell death. Mechanistically, pyroptosis is caused by excessive alarmin S100A9 found in MDS HSPCs and bone marrow plasma. In addition, similar to somatic gene mutations, S100A9-induced signals activate NADPH oxidase (NOX) and increase the level of ROS, which initiate cation influx, cell swelling, and β-catenin activation. It is worth noting that downregulation of NLRP3 or caspase-1, neutralization of S100A9, and drug inhibition of NLRP3 or NOX inhibit MDS cell pyroptosis, ROS production, and nuclear β-catenin activity, which is sufficient to restore effective hematopoiesis [34]. Another study suggested that the expression of S100A9 is increased in MDS patients, which promotes the aging phenotype of bone marrow stromal cells through the Toll-like receptor 4 (TLR4) signaling pathway, the formation of the NLRP3 inflammasome and IL-1β secretion [35], in line with the above findings. Therefore, alarmins and founder gene mutations in MDS form a common redox-sensitive inflammatory loop, providing potential new methods for treatment.

## Research progress on pyroptosis in multiple myeloma

Multiple myeloma (MM) is malignant proliferative plasma cell disease. Some studies have shown that cell death is related to the treatment outcome and prognosis of MM. Protein arginine methyltransferase 5 (PRMT5) is a histone methyltransferase involved in the growth of a variety of hematological malignant tumor cells. Through bioinformatics analysis, we found that the expression of PRMT5 in MM was significantly upregulated [36]. In addition, we found that the expression of CASP1 and PRMT5 showed a negative correlation. CASP1 initiates the pyroptosis pathway by cleaving GSDMD and hydrolyzing the precursors of the inflammatory cytokines IL-1b and IL-18, which is a key factor distinguishing pyroptosis from apoptosis and necrosis [37••]. Silencing the expression of PRMT5 can upregulate the expression of N-GSDMD, IL-1b, and IL-18, promote the expression of CASP1, and induce apoptosis in MM cells. Furthermore, high expression of PRMT5 and low expression of CASP1 are associated with low overall survival in MM. Altogether, these findings provide a mechanism by which PRMT5 regulates pyroptotic cell death by silencing CASP1 in MM [36].

The proto-oncogene MYC is dysregulated in approximately 70% of human cancers and is overexpressed in MM [38, 39]. As a pleiotropic transcription factor, MYC regulates gene expression and multiple pathways involved in cell growth, proliferation, metabolism, and apoptosis. In the pathogenesis of MM, the activation of MYC is the result of translocation, rearrangement, and/or modification of the MYC gene, so targeting MYC is an attractive strategy for MM therapy [40–42]. The transcriptional regulation of MYC involves multiple promoters, enhancers, and transcriptional initiation sites. The upstream P1 promoter of c-MYC contains nuclease hypersensitive element (NHE) III, which controls 85–90% of the transcriptional activity of the gene. The NHE III region contains a noncoding chain rich in purine and forms an atypical Hoogsteen-bonded structure called the G-quadruplex (G4). G4 acts as a transcriptional suppressor element that can regulate MYC transcription by ligand-mediated G4 stability. In general, G4 structures are in dynamic equilibrium with normal double-stranded structures and do not naturally form at high frequencies to block transcription, partly because they can be broken down by helicases. The development of small molecular compounds that stabilize MYC G4 has become a focus of studies developing therapies for MYC-driven tumors [43–45]. Previously, we demonstrated that stabilizing G4 in the MYC promoter region inhibits MYC transcriptional function [45]. Using a small molecular chip assay, we found a selective MYC G4-binding drug with a benzofuran scaffold (D089) that can not only inhibit the expression of MYC in MM cell lines but also selectively induce G1 phase arrest in MYC-driven cancer cell lines containing the MYC G4 sequence. We found that D089 regulates cell cycle progression, senescence, and caspase-1-mediated pyroptosis by inducing activation of typical pathways related to the unfolded protein response, endoplasmic reticulum stress, and inflammation [46].

Multiple prognostic pyroptosis-related gene (PRG) signals for different types of cancers have been identified [16, 47–49]. Through Cox and LASSO analyses, we assessed PRG signals and their possible physiological significance in clinical samples and MM transcriptome data from GEO data and identified 11 characteristics PRG genes related to the prognosis of MM patients: AIM2, CASP1, Elane, GSDMB, GSDMC, IL1B, NLRP1, GZMB, IL1a, CHMP7, and CYCS [50]. Doxorubicin (DOX) is widely used as a drug for MM, it is usually used in combination with other adjuvant drugs [51] and remarkably induces pyroptosis through caspase-3-induced GSDME fragments and leads to pyroptotic cell death [52, 53]. In recent years, studies have shown that the DOX-induced cell death pathway involves caspase-3-mediated activation of GSDME, suggesting that GSDME may be a potential drug treatment target. However, there are few studies on the mechanism of MM pyroptosis induced by DOX. Q-VD-OPH is a pan-caspase inhibitor that participates in caspase-dependent apoptosis and inhibits many caspases (such as caspase-1, caspase-3, caspase-7, caspase-8, caspase-9, caspase-10, caspase-11, and caspase-12) for extended durations and does not show cytotoxicity even at very high concentrations [54]. Flow cytometry showed that the cell death induced by DOX significantly decreased after the addition of Q-VD-OPH. Additionally, the protein level of cleaved GSDME in DOX-treated MM cells confirmed that DOX triggered pyroptotic cell death. In addition, after Q-VD-OPH treatment, the level of GSDME-N induced by DOX treatment decreased, suggesting that Q-VD-OPH inhibits the pyroptosis induced by DOX to some extent. These findings suggest that an understanding of the genes associated with pyroptosis may provide new insights for the development of future anti-MM therapies [50].

## Research progress on pyroptosis in lymphoma

The role of pyroptosis in lymphoma has been studied. Some studies have shown that BAFF supports the survival and dynamic balance of B cells by activating NF-κB pathway. The binding of BAFF to the BAFF receptor triggers the initiation and activation of the NLRP3 inflammasome in primary B cells and B lymphoma cell lines. Activation of the NLRP3 inflammasome induced by BAFF increases the expression of NLRP3 and IL-1β, activates caspase-1, increases the secretion of IL-1β, and leads to cell death. Mechanistically, BAFF activates the NLRP3 inflammasome by enhancing the binding of CIAP-TRAF2 to NLRP3 inflammasome components and thus induces Src activity-dependent ROS production and potassium ion efflux. Stimulation of B cell receptor (BCR) in the LYN signaling pathway can inhibit BAFF-induced Src activity and attenuate BAFF-induced activation of the NLRP3 inflammasome. These findings reveal another function of BAFF in B cell homeostasis, which is related to BCR activity [55]. Sesamin is a lignan compound in plants that has a variety of pharmacological effects. In a mouse model of T cell lymphoma, we found that sesamin significantly inhibited the proliferation of EL4 cells by inducing apoptosis, pyroptosis, and autophagy. After sesamin treatment, autophagy of EL4 cells preceded apoptosis and pyroptosis. Blocking autophagy inhibited apoptosis and pyroptosis of EL4 cells treated with sesamin, suggesting that sesamin promotes apoptosis and pyroptosis through autophagy pathways, thus enhancing the effect against T cell lymphoma in mice, which provides a theoretical basis for the development of new antitumor drugs for the treatment of T cell lymphoma [56] (Table 1).

**Table 1:** Research progress on pyroptosis in hematological malignancies

Type of hematologic malignancy	Key findings	Reference
AML	Dpp8/9 inhibitor activates the caspase-1 signaling pathway, leading to the cleavage and activation of GSDMD, and inhibits AML progression	[19]
	Administration of BAY-299 increases the expression of pyroptosis-related genes, and activation of caspase-1, caspase-4, caspase-5, or caspase-11 can induce GSDMD-related pyroptosis	[20]
	Curcumin can induce the expression of AIM2, IFI16, and the NLRC4 inflammasome in U937 leukemia cells by upregulating the expression of the ISG3 transcription factor complex and then activate caspase-1, promoting the cleavage of GSDMD and inducing cell death	[24]
	Ardisianone can induce the cleavage of caspase-1, caspase-5, caspase-8, and GSDMD and increase the expression of HMGB1 protein, promoting pyroptosis of HL60 cells	[25]
	Tp92 induces atypical pyroptosis of THP-1 cells by promoting activation the caspase-1 pathway	[27]
	NSA directly binds to the pyroptotic pore-forming protein GSDMD, inhibiting GSDMD oligomerization and pyroptotic cell death	[28]
MDS	Downregulation of NLRP3 or caspase-1, neutralization of S100A9, and drug inhibition of NLRP3 or NOX inhibit MDS cell pyroptosis	[34]
	The expression of S100A9 is increased in MDS patients and promotes the aging phenotype of bone marrow stromal cells through the TLR4 signal pathway, the formation of the NLRP3 inflammasome and IL-1β secretion	[35]
MM	Silencing the expression of PRMT5 can upregulate the expression of N-GSDMD, IL-1b and IL-18, promote the expression of CASP1, and induce MM cells to pyroptosis	[36]
	D089 regulates cell cycle progression, senescence and caspase-1-mediated pyroptosis by inducing typical pathways involved in unfolded protein response, endoplasmic reticulum stress and inflammation	[46]
	Q-VD-OPH inhibits pyroptosis induced by DOX	[54]
NHL	Sesamin significantly inhibits the proliferation of EL4 cells by inducing apoptosis, pyroptosis, and autophagy; after Sesamin treatment, autophagy of EL4 cells preceded apoptosis and pyroptosis	[56]

*AML*, acute myeloid leukemia; *MDS*, myelodysplastic syndrome; *MM*, multiple myeloma; *NHL*, non-Hodgkin lymphoma; *GSDMD*, gasdermin-D; *NSA*, necrosulfonamide; *TLR4*, Toll-like receptor 4; *PRMT5*, protein arginine methyltransferase 5; *NOX*, NADPH oxidase; *CASP1*, caspase-1; *DOX*, doxorubicin

## Increased understanding of pyroptosis in immune research

Pyroptosis is characterized by cell swelling, lysis, and the release of many proinflammatory factors, including IL-1β, IL-18, ATP, and HMGB1, that mediate the type of inflammatory regulated cell death. Many published articles have reported that tumor cells undergoing pyroptosis recruit tumor-suppressive immune cells [8, 57]. For example, Wang et al. constructed systems to demonstrate that pyroptosis of less than 15% of tumor cells was sufficient to clear an entire tumor graft using live animal models [57]. In addition, another study performed by Zhang et al. illustrated that in the pyroptosis-activated immune microenvironment, CD8 + T cells and natural killer (NK) cells induce pyroptosis of tumor cells via granzyme B (an enzyme capable of cleaving GSDME), establishing a positive feedback loop [8]. Additionally, the researchers showed that GSDME inactivation is an important mechanism used by cancer cells to escape immune attack. Furthermore, CD8 + T and NK cells were demonstrated to trigger tumor clearance through the GSDMB-granzyme A axis, which could be enhanced by IFN-γ [22]. The researchers proved that the expression of GSDMB induced pyroptosis via granzyme A. Altogether, these results indicate that pyroptosis is promoted by NK cells, suggesting that tumors potentially dictate the activation of the respective GSDM-granzyme axis [8, 22, 57].

## Conclusions and perspectives

In this review, we provided the definition, basic characteristics, and developmental history of pyroptosis; briefly explained the mechanism of pyroptosis; and summarized the latest understanding of pyroptosis in hematological tumors. At present, hematological malignant tumors are some of the most common malignant tumors and substantially affect human health. In recent years, with the progress of radiotherapy, chemotherapy, and hematopoietic stem cell transplantation and the emergence of new treatments such as targeted therapy, biotherapy, and cell therapy, the prognosis of patients with hematological tumors has been greatly improved. However, due to various factors, the ultimate survival time of patients with hematological malignant tumors is still very short, and there is an urgent need for new treatment strategies to improve patient survival. Pyroptosis is a form of inflammatory cell death dependent on caspases. Many studies have revealed the mechanisms of pyroptosis and the and potential applications related to pyroptosis in hematological malignant tumors. However, we have just begun to understand the role of pyroptosis in hematological malignant tumors, and there is still a lack of research on the mechanisms. In the future, more in-depth studies of the mechanisms of hematological malignant tumor cells are expected to reveal new treatment strategies.
